# Supplementary material for: A Global Screen for Assembly State Changes of the Mitotic Proteome by SEC-SWATH-MS
Source: Cell Syst. 2020 Feb 26;10(2):133–155.e6. doi: 10.1016/j.cels.2020.01.001 (PMC7042714; doi:10.1016/j.cels.2020.01.001)

Q06547 | GABP1_HUMAN | GABPB1 E4TF1B GABPB GABPB2
Monomer MW [kDa]: 42.483 Monomer expected elution fraction: 48

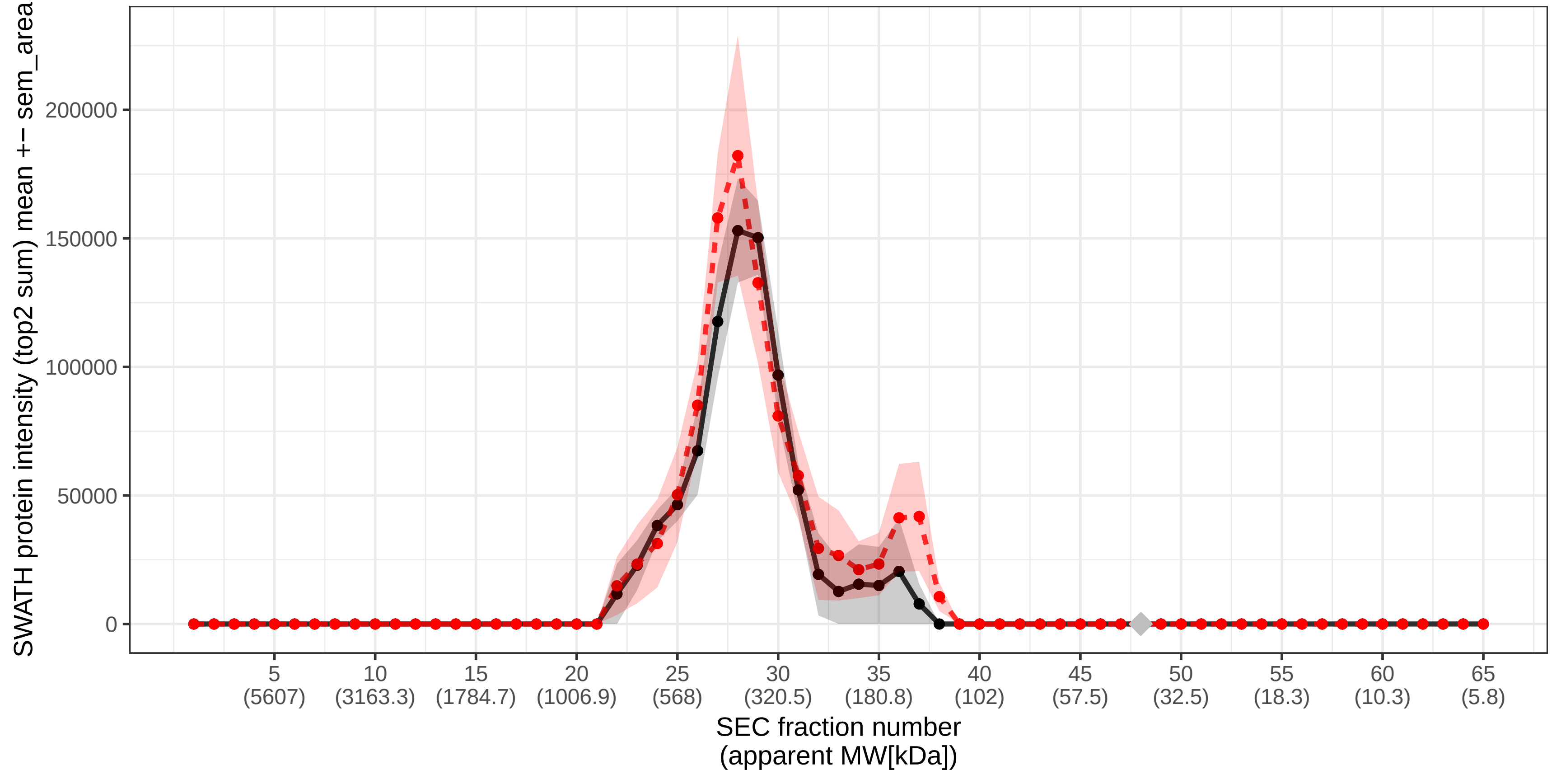

Supplement: Data S1. SEC-SWATH-MS Protein Chromatograms, Related to Figure 1 [file mmc6.zip › SECchrom_Q06547_GABP1_HUMAN_GABPB1_E4TF1B_GABPB_GABPB2.pdf]
